# Mg Alloys Reduce Gastric Cancer and Mediate Therapeutic Management of Antibacterial Function

**DOI:** 10.3390/jfb17010021

**Published:** 2025-12-29

**Authors:** Yonghong Wu, Xiaoyun Lu, Yiwei Wang, Guangyan Liu, Biao Yang

**Affiliations:** 1 School of Medical Technology, Xi’an Medical University, Xi’an 710021, China; wuyonghong@xiyi.edu.cn; 2 Key Laboratory of Biomedical Information Engineering of Ministry of Education, School of Life Science and Technology, Xi’an Jiaotong University, Xi’an 710049, China; luxy05@xjtu.edu.cn; 3 Department of Pathology, Shenyang Medical College, No. 146, Huanghe North Street, Shenyang 110034, China; wangyiwei_symc@163.com; 4 Department of Pathogen Biology, Shenyang Medical College, No. 146, Huanghe North Street, Shenyang 110034, China; liuguangyan@symc.edu.cn

**Keywords:** gastric carcinoma, Mg alloys, anticancer, antibacterial effects, transcriptome

## Abstract

Background: Gastric cancer (GC) represents a significant challenge in global public health, and novel treatment strategies are urgently needed. This study investigated the potential application of Mg alloys for the treatment of GC through preclinical experimentation. Methods: Alloy materials were screened and selected in GC cells using cell viability assays. To uncover the mechanisms by which Mg alloys influence GC, RNA sequencing and qRT-PCR were performed. Mice bearing tumors derived from GC cells were used to assess the potential application of Mg alloys in GC. The antibacterial effects of Mg alloys were evaluated in *Escherichia coli* and *Staphylococcus aureus*. Results: Co-cultures of Mg alloys with MGC-803 cells resulted in inhibition of cell viability. RNA sequencing revealed differential mRNA expression and we validated the gene expression changes. Moreover, Mg alloy wire implantation effectively displayed that the inhibition rate of relative tumor volume reached 42.86% in tumor-bearing mice. Additionally, Mg alloys inhibited bacterial growth of two types of pathogenic bacteria, with an antibacterial rate of approximately 70%. Conclusions: Our results indicate that Mg alloys represent a novel therapeutic resource for clinical applications against GC.

## 1. Introduction

Gastric cancer (GC) is a common type of clinical tumor, with the incidence and mortality rates both ranking fifth among malignant tumors worldwide [1]. Because of the limitations in early diagnosis technology, most GC patients are diagnosed with local advanced or metastatic disease, resulting in generally poor clinical prognoses [2]. Currently, the treatment of GC primarily involves radiotherapy, chemotherapy, and surgery. While these modalities are effective, radiotherapy and chemotherapy lack targeting of tumor tissues and may cause toxic and side effects in normal cells. Therefore, the development of novel technologies for diagnosis, and therapeutic approaches to the treatment of GC to extend patient survival and improve quality of life represent critical areas of research.

To improve the prognoses for GC in clinical settings and address this significant public health challenge, researchers have investigated the use of novel materials as adjunctive therapeutic agents to prevent postoperative tumor recurrence [3,4]. In recent years, numerous studies have systematically explored the targeting mechanisms and application potential of various therapeutic strategies, including metal-based nanocarriers, metal-organic frameworks, mesoporous silica nanocarriers, carbon-based nanomaterials, quantum dots, and green-synthesized inorganic nanocarriers [5,6,7]. However, the biological toxicity and long-term therapeutic effects of these materials are unknown, and their ability to adapt to the human physiological environment is limited, thereby restricting their clinical application.

Research has demonstrated that magnesium (Mg) alloys exhibit favorable biocompatibility and mechanical properties [8]. The degradation products of Mg alloys mainly include Mg ions (Mg^2+^), hydroxide ions (OH^−^), and hydrogen gas (H2). Increasing evidence has shown that Mg alloys possess significant application potential. For patients requiring stent implantation and subsequent secondary interventions, the biodegradable nature of Mg alloys can mitigate the need for additional surgical procedures, thereby reducing patient discomfort and procedural risks. Studies have also demonstrated that Mg alloys have anti-tumor properties. The mechanisms of the anti-tumor activity of Mg alloys and their biodegradation products have been clarified in several tumor studies [9,10]. The therapeutic application of Mg alloys in cancer is currently in the early stages, and the effect of Mg alloys on gastrointestinal malignancies is still poorly understood.

In this study, we systematically evaluated the therapeutic potential of Mg alloys in GC, a highly prevalent and clinically aggressive malignant cancer, in MGC-803 cells and tumor-bearing mice. RNA sequencing (RNA-seq) was performed to elucidate the molecular mechanisms underlying the effects of Mg alloys on tumor dynamics. This study provides valuable insights into the mechanisms by which Mg alloys exhibit anti-tumor activities, offering novel perspectives and a strategic approach for future technological advancements and clinical translation, which is of great importance for improving the treatment of patients with GC.

## 2. Materials and Methods

### 2.1. Sample Preparation and Sterilization

High-purity Mg ingots (99.98% purity) were hot-extruded into rods and subsequently drawn into wires with diameters of 5 mm and 1 mm. A laminar structure was further fabricated from rods of 10 mm and 1 mm diameters using a combined process of multi-pass cold drawing and annealing at room temperature. The wire was used for in vivo tumor-bearing mice, and a laminar structure was used for in vitro experiments. The alloy materials were totally immersed in 75% alcohol (5 min) and were sterilized by irradiating the wire surface with ultraviolet (UV) for 20 min. Surface morphology of the alloys was confirmed under the scanning electron microscope (SEM), and the materials were soaked in Hank’s liquid for 24 h.

### 2.2. Cell Culture

The cells utilized in this experiment were MGC-803, a human gastric carcinoma cell line, obtained from the Cell Bank of the Chinese Academy of Sciences (CBCAS) in Shanghai, China. The cells were cultured in High-DMEM medium supplemented with 10% fetal bovine serum (Hyclone, Logan, UT, USA) and 1% penicillin-streptomycin (Hyclone, USA), and maintained at 37 °C in an atmosphere containing 5% CO_2_.

Cells were seeded at a density of 1.0 × 10^4^ per well in triplicate within 96-well plates. Subsequently, the laminar structure composed of Mg wires and titanium (Ti) alloy (10 × 1 mm) was placed into 1000 μL of medium within six-well plates. Following the 24 h exposure period, we collected the exposed culture medium to conduct a cell viability assay.

### 2.3. Cell Viability

Cell viability was evaluated using CellTiter 96 AQueous One Solution Reagent (Promega, Madison, WI, USA) in accordance with the manufacturer’s instructions. Following 24 h treatment with the exposed culture medium, absorbance readings were obtained using the Infinite M200 Pro Reader (TECAN, Männedorf, Switzerland) at a wavelength of 490 nm after a duration of 2 h. All assays were conducted in triplicate across three independent experiments.

### 2.4. Gene Expression Data

Transcriptome high-throughput sequencing was conducted at Lianchuan Biochip Company (Hangzhou, China). The FPKM (Fragments Per Kilobase of exon model per Million mapped reads) values were analyzed for quantitative normalization, derived from different genes across two samples using Cufflinks. Differentially expressed genes were identified based on a Fold Change ≥ 2 and a False Discovery Rate (FDR) ≤ 0.05. The Gene Ontology (GO) project aims to describe the attributes of genes and gene products (http://www.geneontology.org, accessed on 25 November 2023) and pathway analysis serves as the Kyoto Encyclopedia of Genes and Genomes (KEGG) pathways (http://www.genome.jp/kegg/, accessed on 25 November 2023).

### 2.5. RNA Extraction and Quantitative Real-Time PCR

Performing quantitative real-time PCR (qRT-PCR) to assess the fold changes of candidate genes, we utilized an ABI Prism 7500 (Applied Biosystems, Carlsbad, CA, USA) along with SYBR Master Mix (Applied Biosystems, Carlsbad, CA, USA), adhering strictly to the manufacturer’s instructions. Total RNA was extracted and subsequently treated with DNase I before being reverse transcribed (Promega, Madison, WI, USA). The primer pairs employed for qRT-PCR are detailed in Table 1. The results of the PCR analysis of mRNA levels were normalized against GAPDH expression, and relative expression levels were calculated using the 2-ΔΔCt method.

### 2.6. Protein Network Construction

This study utilized protein–protein interaction data sourced from the STRING database to construct an integrated interaction network of Mg alloy-mediated GC-related proteins. This network integrated and weighted the information from multiple evidence sources, including activation, inhibition, binding, catalysis, reaction, and gene expression, etc. [11]. To ensure reliability, only high-confidence interactions with a combined score higher than 0.7 were retained for further analysis.

### 2.7. Animals and the Tumor Xenograft Growth In Vivo

BALB/c nude mice (16~20 g) were purchased from Vital River. CP (Beijing, China). All mice were acclimatized for two weeks in Laboratory Animal Care. To easily measure and re-create gastric tumors in various mouse strains, an allografted tumor model was created. We firstly inoculated subcutaneously into the flanks of BALB/c nude mice with the MGC-803 cells. When the tumor grew to a diameter of 10 mm, we collected the tumor tissue and then re-implanted it into the flanks of the BALB/c nude mice. After the tumor grew to an appropriate size, and they were randomly divided into a Ti alloy group (*n* = 6) and Mg alloy group (*n* = 6) according to the random number table method. The primary operational components of this study encompass metal implantation and tumor measurement. We conducted metal implantation at the outset of the grouping process, followed by tumor volume assessments on day 0, day 5, and day 10, respectively.

**Figure A1:**
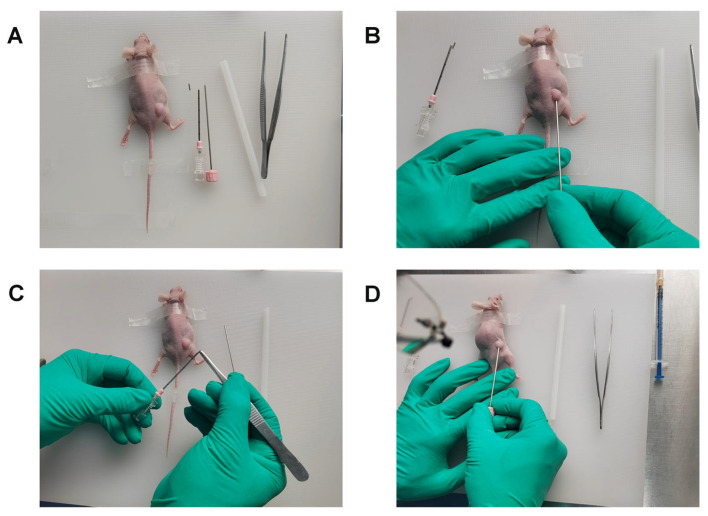
Alloy material implantation scheme. (**A**) Preoperative Preparation. (**B**) Tumor puncture procedure. (**C**) Preparation for metal material implantation. (**D**) Metallic Material Implantation Surgery.

After establishing the tumor-bearing mice, we performed the material implantation as shown in Appendix A Figure A1. The skin was disinfected with iodophor, and the alloy material was loaded into the implantation device. The material was then precisely implanted into the tumor via percutaneous puncture. Postoperatively, the surgical site was disinfected for three days.

### 2.8. Histopathological Analyses

Each group of mice was sacrificed on day 20, and we ablated major organs to evaluate biological safety. All tissues were fixed in 4% paraformaldehyde for 2 weeks and then embedded in paraffin and cut into 5 μm sections. All sections were next prepared for hematoxylin and eosin (H&E) staining.

### 2.9. Antibacterial Test

The bacterial strains of *Escherichia coli* (*E. coli*) and *Staphylococcus aureus* (*S. aureus*) were preserved in our laboratory at the Key Laboratory of Environmental Pollution and Microecology, Liaoning. The strains were cultured on Brain Heart Infusion (BHI) agar plates at 37 °C for 12 h. A single colony was selected and subsequently cultured in BHI liquid medium for an additional 12 h. The ninth generation of bacteria was diluted to a concentration of 1 × 10^4^ colony-forming units (CFU)/mL using Hank’s solution. Samples made from Mg alloys were immersed in Hank’s solution at 37 °C for a duration of 24 h. Two types of soaking solutions, each containing 2 mL, were combined with a corresponding volume of bacterial suspension (2 mL), followed by co-culturing at 37 °C for 6 h. After ensuring that the mixed suspension was thoroughly homogenized, aliquots of 100 μL from the suspensions were plated onto BHI agar plates in triplicate and incubated for another 12 h. The antibacterial efficacy was evaluated using the following formula: Antibacterial rate (%) = [(Nc − Ne)/Nc] × 100%, where Nc represents the CFUs of bacteria in the control group, and Ne denotes the CFUs of bacteria in the experimental group.

### 2.10. Statistics

Data were analyzed using GraphPad Prism 6 (GraphPad Software, San Diego, CA, USA). Results are presented as mean ± standard deviation (SD) from three independent experiments. Group differences were assessed by one-way ANOVA. For comparisons between two groups, statistical significance was determined using an unpaired, two-tailed Student’s *t*-test. A *p* value < 0.05 was considered statistically significant.

## 3. Results

### 3.1. Degradation Performance of Mg Alloys

**Figure 1:**
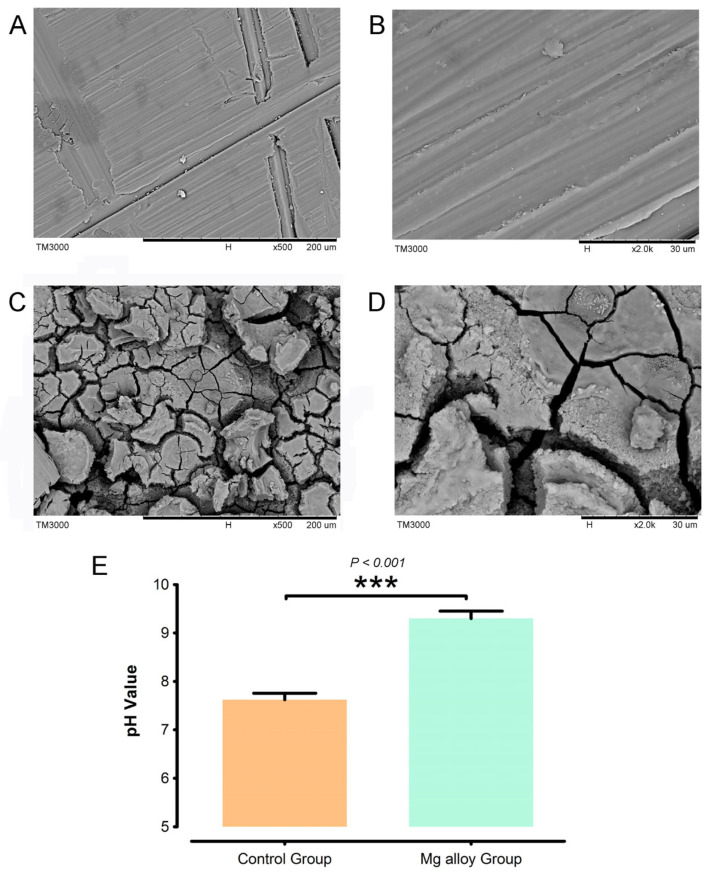
Degradation performance of Mg. (**A**) The morphological characteristics of Mg alloys at 500× and (**B**) 2000×. (**C**) The degradation morphology of Mg alloys at 500× and (**D**) 2000×. (**E**) pH values were evaluated. The experiments were performed at least three times. *** *p* < 0.001 compared with control group.

To investigate the effects of alloy materials in a liquid environment we conducted a surface morphology analysis of Mg alloys using SEM (Figure 1) and Energy Dispersive Spectroscopy (EDS) analysis (Table 2). Initially, we examined the materials that had not been immersed in liquid, as well as those that had been soaked in Hank’s solution for one day (Figure 1A–D). The metal samples submerged in Hank’s solution exhibited significant surface deposits and a marked reduction in the detection of Mg elements. Furthermore, it was observed that pH value gradually increased with prolonged immersion time of Mg alloys (Figure 1E).

### 3.2. Effects of Mg Alloys Exposure on the Proliferation of MGC-803

**Figure 2:**
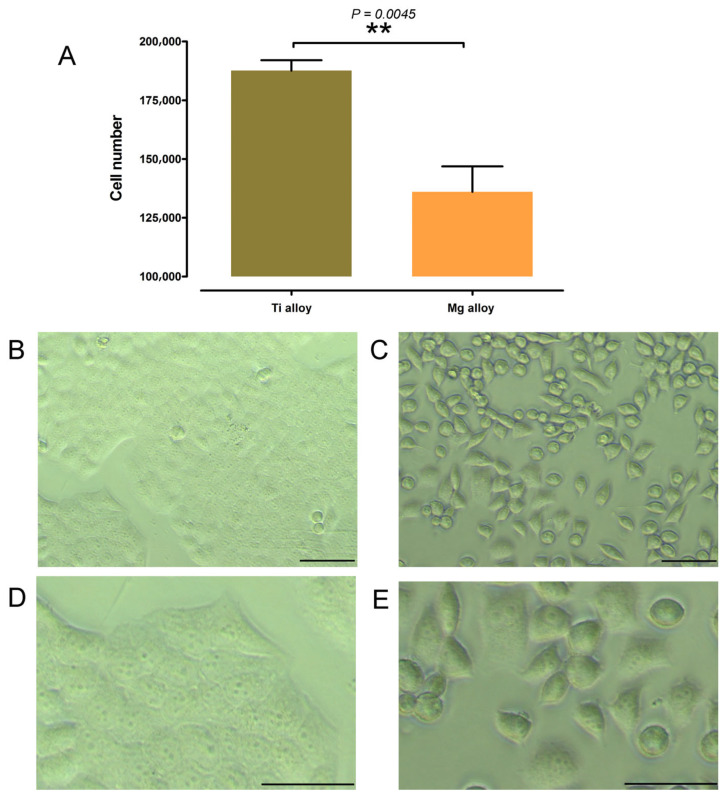
Effects of Mg alloy exposure on cell proliferation in MHC-803 cell lines. (**A**) Cell viability 24 h. (**B**) Morphology after 24 h of Ti alloy-exposed culture environment, 10×. (**C**) Morphology after 24 h of Ti alloy-exposed culture environment, 10×. (**D**) Morphology after 24 h of Ti alloy-exposed culture environment, 40×. (**E**) Mg alloy-exposed culture supernatants group, 40×. Scale bar, 20 μm. Data represent the means ± SD of three independent experiments. ** *p* < 0.01 compared with control group.

To investigate the differences in the proliferation of MGC-803 cell lines induced by culture supernatants exposed to Mg alloys, the cell proliferation assays were conducted over a 24 h interval. In comparison to the Ti alloy group, the Mg alloy-exposed culture supernatants demonstrated significantly lower cell viability and proliferation (Figure 2). Collectively, our findings indicated that MGC-803 cells exhibited reduced population levels following exposure to Mg alloys.

### 3.3. Differential mRNA Expression Profiling Following Mg Alloy Exposure on MGC-803 Cells

**Figure 3:**
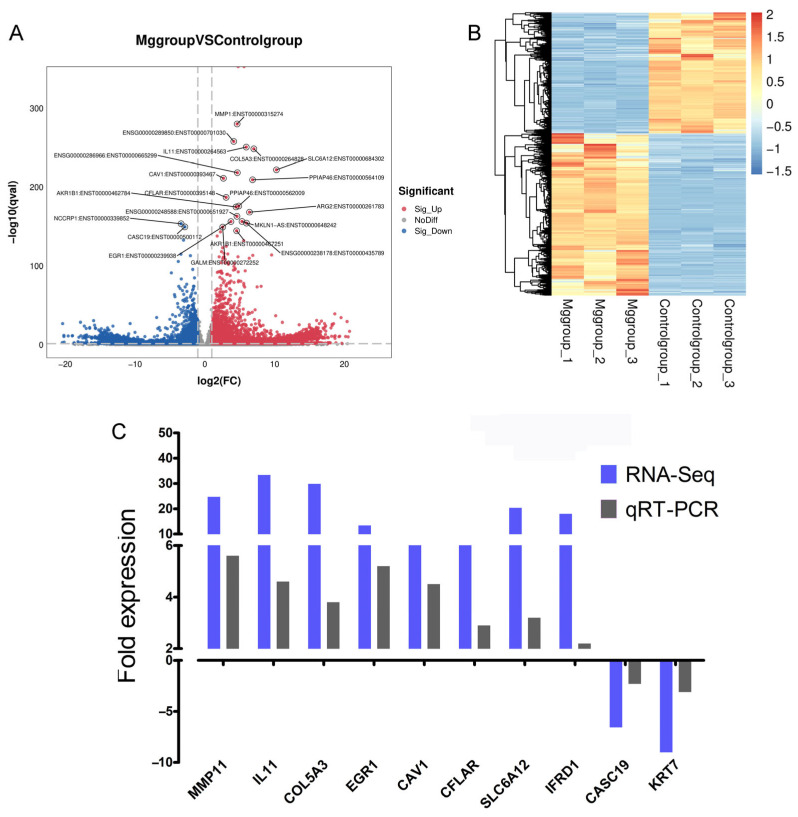
Differential mRNA expression profiling. (**A**) Transcripts volcano plot of DEGs. (**B**) Heatmap of DEGs. (**C**) Correlation of transcriptional changes assayed by RNA-seq with those assayed by qRT-PCR.

Through RNA Seq analysis, an average of 10 G data was generated for each sample, with a total of 86,120 genes detected. As shown in Figure 3, we obtained a total of 14,832 differentially expressed genes (DEGs), of which 8537 were upregulated and 6295 were downregulated. At the same time, in order to verify the expression level of mRNA, nine significantly different genes were randomly selected from the results of RNA-Seq to complete qRT PCR experiments.

To validate the expression levels of mRNA, genes exhibiting significantly different expression levels were randomly selected from the microarray data for verification through qRT-PCR, utilizing cDNA derived from two experimental samples. Among these significant DEGs, six were found to be upregulated (MMP1, IL11, COL5A3, SLC6A12, EGR1, and IFRD1) while two were downregulated (CASC19 and KRT7). GAPDH was used as an internal control to normalize target RNA. The results obtained from qRT-PCR analysis were corroborated by experimental findings, demonstrating statistically significant differences that aligned with the trends observed in RNA-seq (Figure 3C).

### 3.4. GO and Pathway Analysis

**Figure 4:**
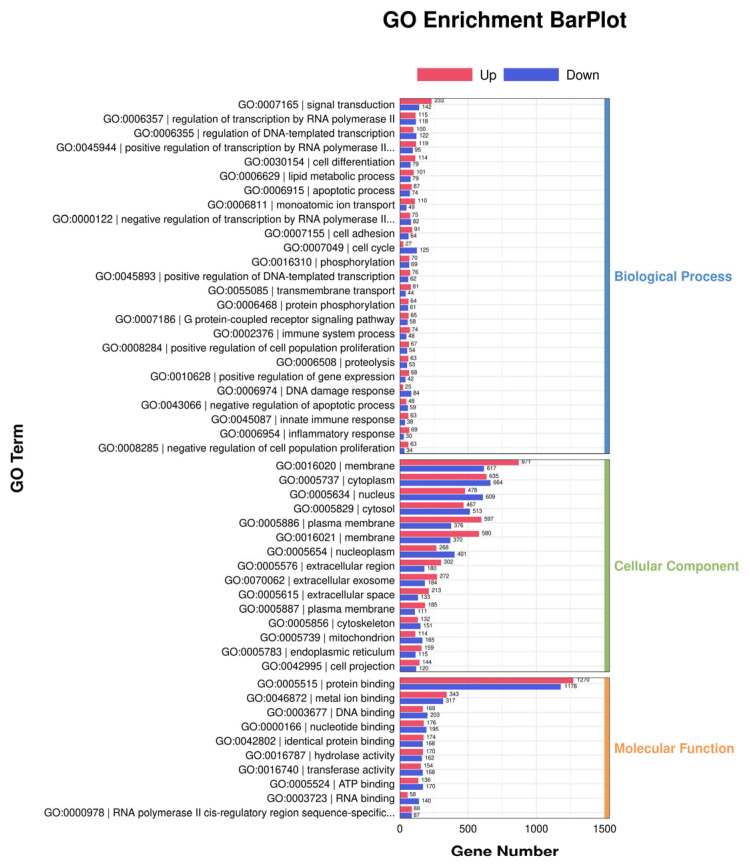
GO analysis of differentially expressed genes.

The functions associated with the DEGs are presented in Figure 4. We found that the most common biological processes targeted by signal transduction were regulation of transcription by RNA polymerase, DNA templated transcription, positive regulation of transcription by RNA polymerase, and cell differentiation. Our findings indicated that the most common cellular components targeted by these transcripts encompass the membrane, cytoplasm, nucleus, cytosol, and plasma membrane. The results indicated that the most common molecular functions targeted by protein binding were metal ion binding, DNA binding, nucleotide binding, and identical protein binding.

**Figure 5:**
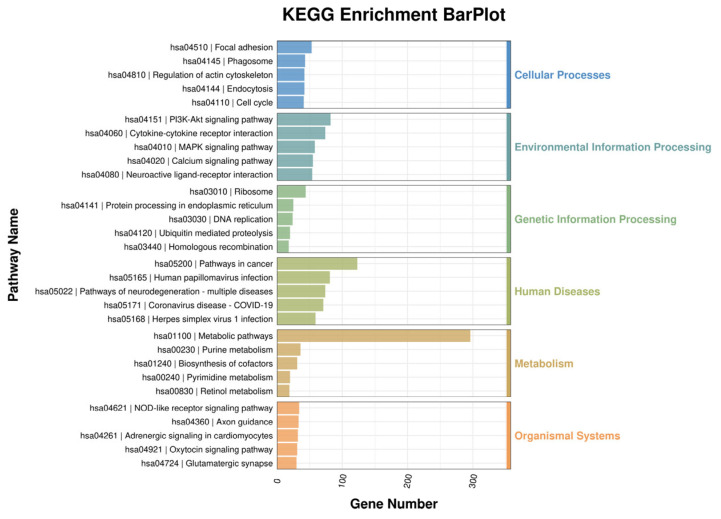
KEGG pathway analysis for differentially expressed genes (DEGs).

Pathway analysis is illustrated in Figure 5. We identified that the most prevalent functions of the transcripts from these DEGs included protein processing in the cellular processes, environmental information processing, genetic information processing, human diseases, metabolism, and organismal systems.

### 3.5. Network Analysis of Genes Affected by Mg Alloy-Exposed MGC-803 Cells

**Figure 6:**
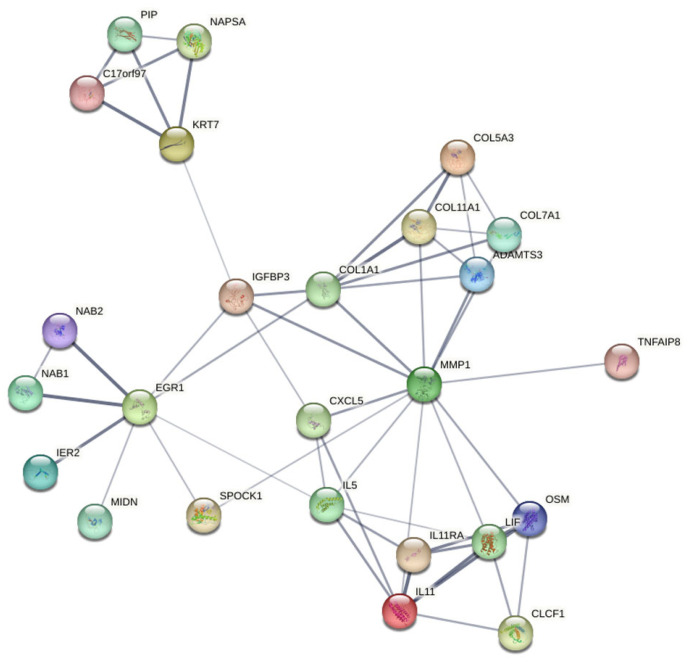
Interactive gene network view of Mg alloy-exposed MGC-803 cells.

In our study, we analyzed selected genes from the STRING database that revealed significant associations. The results of RNA sequencing in Mg alloy-exposed MGC-803 cells indicated enhancement of a network comprising interacting proteins (Figure 6). Additionally, we expanded our analysis based on these findings to include related genes based. Within the constructed network, MMP1, KRT7, EGR1, and IL11 played crucial roles, likely due to the cell lines representing invasive and proliferative cancers. Interactions with combined scores exceeding 0.4 were considered high confidence; therefore, we focused on interactions with combined scores greater than 0.7 for further analysis.

### 3.6. MMP1, IL11, EGR1, and KRT7 Expression Correlates with GC Prognosis

**Figure 7:**
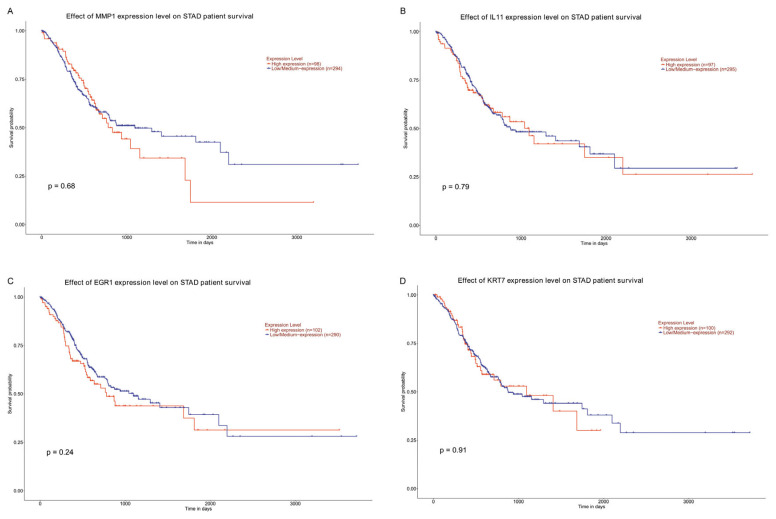
DEG expression correlates with GC prognosis. (**A**) MMP1 expression correlates with GC prognosis. (**B**) IL11 expression correlates with GC prognosis. (**C**) EGR1 expression correlates with GC prognosis. (**D**) KRT7 expression correlates with GC prognosis.

In the current study, to elucidate the clinical relevance of DEGs, we tested the potential association between MMP1, IL11, EGR1, and KRT7 expression and clinicopathological features in TCGA data (Figure 7). We then assessed the relationship between differential gene expression level and prognosis in STAD patients by using Kaplan–Meier analysis. STAD patients with high MMP1 and IL11 expression levels had significantly reduced overall survival time than those with low expression levels. STAD patients with low EGR1 and KRT7 expression levels had significantly reduced overall survival time than those with high expression levels.

### 3.7. Effect of Mg Alloy Implantation on Subcutaneous Gastric Tumors

**Figure 8:**
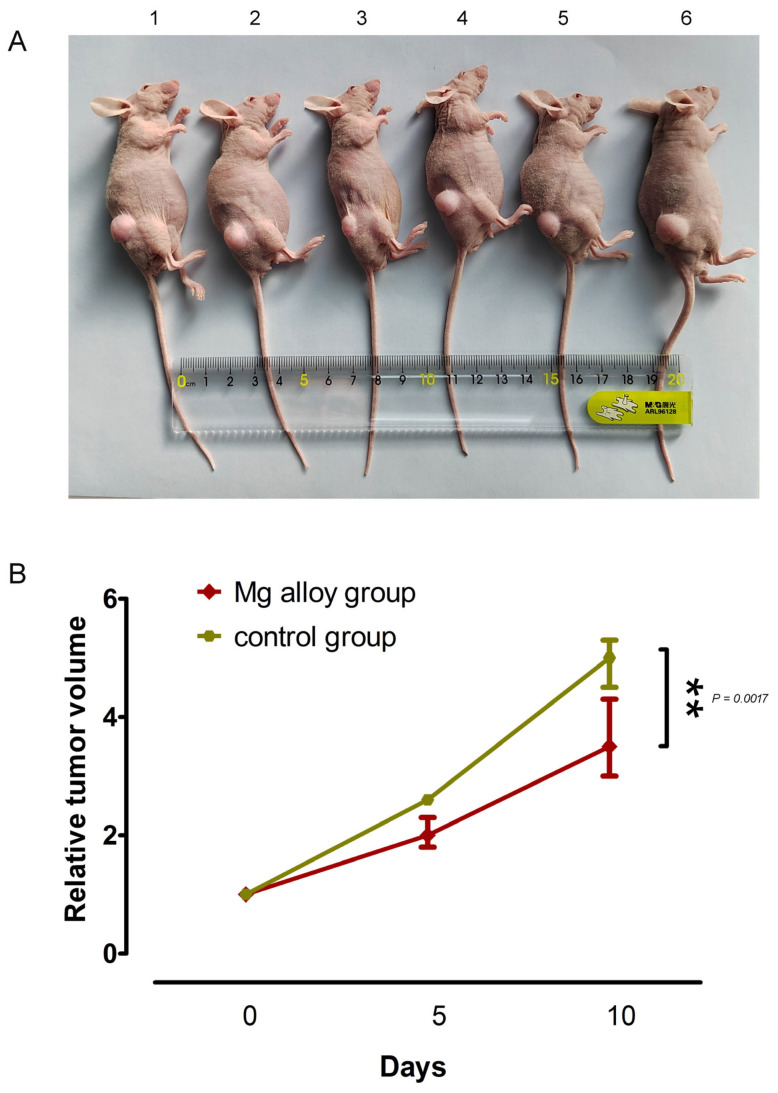
Gastric tumors in tumor-bearing mice implanted with metallic wires. (**A**) 1–3, Mg alloy implantation group. (**A**) 4–6, control group. (**B**) Tumor volume curve of tumor-bearing mice. *N* = 3. ** *p* < 0.01 compared with control group.

To investigate the effects of alloy wires on gastric tumors in mice, this study further assessed the inhibitory effects of Mg alloys on GC growth in vivo. Six Mg alloy wires (1 × 3 mm) were implanted into tumor tissue in the experimental group, while a control group receiving titanium alloy wires was established to account for mechanical injury caused by surgical implantation and physical stimulation by the implanted material. The results are presented in Figure 8. Our results showed that after 10 days of implantation, the volume of xenograft tumors in the Mg wire group showed significant differences with the Ti wire implantation group (*p* < 0.001), and the inhibition rate of relative tumor volume reached 42.86%. Obviously, the results suggested that Mg alloy wire implantation could effectively reduce the volume of subcutaneous GC.

**Figure 9:**
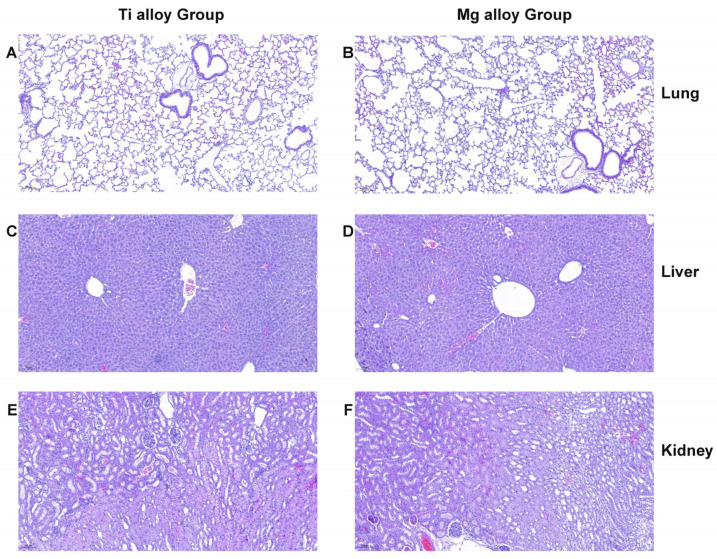
Toxicity analysis of H&E staining images of the main organs of mice implanted with different metal wires at 30 days of implantation. (**A**) The morphological characteristics of lung in the Ti alloy group. (**B**) The morphological characteristics of lung in the Mg alloy group. (**C**) The morphological characteristics of the liver in the Ti alloy group. (**D**) The morphological characteristics of liver in the Mg alloy group. (**E**) The morphological characteristics of kidney in the Ti alloy group. (**F**) The morphological characteristics of kidney in the Mg alloy group.

Additionally, histopathological analyses were conducted on major organs to assess the biological safety of Mg alloy wire implantation. Compared to the Mg wire group, histological examination of key organs following Mg alloy wire implantation revealed no abnormalities upon H&E staining, as illustrated in Figure 9.

### 3.8. Mg Alloys Inhibited Growth in Three Types of Pathogenic Bacteria

**Figure 10:**
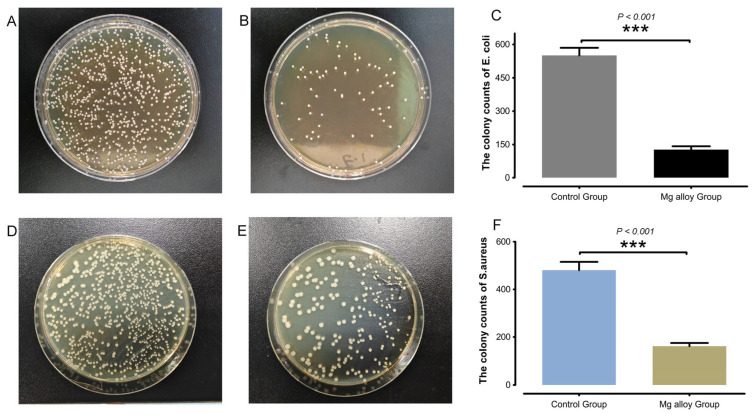
The antibacterial images and antibacterial rates of pure Ti and Mg alloys. (**A**) Mg alloy-exposure group. (**B**) Ti-exposure group. (**C**) The colony counts of *E. coli*. (**D**) Mg alloy exposure group. (**E**) Ti exposure group. (**F**) The colony counts of *S. aureus*. *** *p* < 0.001 compared with control group.

As shown in Figure 10, the antibacterial images suggested that Mg alloys inhibited growth in three types of pathogenic bacteria. The colony counts of *E. coli* and *S. aureus* in the Mg alloy group showed a certain degree of inhibitory effect. The antibacterial rates were accurately determined, with inhibition rates of 77.13% against *E. coli* and 66.46% against *S. aureus*. Our results indicated that Mg alloys exhibit high antibacterial activity.

## 4. Discussion

GC is a malignant disease that seriously threatens human health. As a result of its frequent diagnosis at an advanced stage, the mortality rate of GC is very high [12]. Although traditional therapies such as chemotherapy and molecular targeted therapy have improved patient prognosis, the overall efficacy remains limited, and there is still a need for improvement in patient survival rates. Therefore, the development of new treatment strategies is critical to achieve precise and effective control of GC.

Despite the availability of treatments, the complete removal of tumors is challenging. Cancer recurrence is the result of cells changing their phenotypes through the ability to adapt to microenvironmental stress through evolutionarily conserved mechanisms that enable survival in response to starvation or diapause [13]. Cancer cells with dynamic reprogramming capabilities adapt to different stresses, evade immune surveillance, and disrupt host tissue biology to support tumor regeneration [14]. Metastasis is the main cause of death in patients with GC, but current chemotherapy regimens only target tumor growth and lack the ability to inhibit the invasive and metastatic capabilities of cancer cells. This highlights the necessity of finding new anti-metastatic drug approaches.

In the present study, we co-cultured the Mg alloys with GC cells and found that this exhibited an inhibitory effect on proliferation in vitro. Mg alloys also inhibited tumor growth in vivo in mice bearing tumors derived from GC cells. Several differentially expressed genes were identified by RNA-seq, such as MMP1, IL11, EGR1, and KRT7. These genes encode proteins that activate pathways that regulate inflammation and the tumor microenvironment (TME), stimulate damage to GC cells, and induce cell death. Anastomosis nails replace the traditional manual sutures of the gastrointestinal tract and are widely used for tissue anastomosis and repair after tumor resection in different organs, such as the stomach, intestine, and esophagus. Taken together, these findings support the use of Mg alloys as a material for surgical anastomosis nails in GC patients because these alloys may function to inhibit the growth of remaining tumor cells and prevent micro-metastasis.

MMPs are a crucial medium for tissue remodeling. They are also dysregulated in cancer, playing a multi-dimensional role in tumor development by targeting proteolytic extracellular matrix components [15,16]. Their mechanism of action involves directly promoting tumor invasion and metastasis, remodeling the TME, regulating immune responses, and mediating treatment resistance. Increased expression of matrix metalloproteinase-1 (MMP1), a member of the MMP family, is associated with poor survival outcomes, including disease progression and metastasis, in breast cancer [17]. However, from a holistic perspective, protein expression exerts dual functional roles. By degrading tissue stiffness, MMPs may concurrently enhance the therapeutic efficacy of drugs to a certain extent. In our study, RNA-seq revealed high expression of MMP1 in vitro. This indicates that as a result of the cytotoxic effect of Mg alloys, the compactness of tumor tissue was altered, facilitating the immune environment’s action on tumor cells.

IL11, a member of the IL6 family, is a critical lynchpin between inflammation and cancer and is involved in inflammation and immune responses. IL11 also plays a role in tumorigenesis by altering angiogenesis and metastasis [18]. IL11 is an important contributor to cancer biology and may serve as a stromal cell-derived pleiotropic cytokine with profibrotic and cellular remodeling properties in disease progression [19]. These inflammatory responses activate transcription factors implicated in cancer development and progression, including the signal transducer and activator of transcription 3 (STAT3) hypoxia-inducible factor 1 alpha (HIF1α), and nuclear factor-κB (NF-κB), whose activity controls the further production of inflammatory mediators [20]. The extracellular pH of the TME is low, and the accumulation of OH^−^ increases the local pH surrounding Mg implants and interferes with the TME. The Warburg effect also raises intracellular pH value, making it tend to be alkaline. This is conducive to anaerobic glycolysis in tumor cells and accelerates cell proliferation. It can also increase tumor cell resistance to hypoxia, apoptosis, and other processes. HIF-1 affects the proliferation and invasion of tumor cells by regulating the expression of glycolysis-related enzymes and promoting glycolysis [21]. Therefore, those alterations play an essential role in the plasticity of the TME and promote a pro-tumorigenic microenvironment that favors tumorigenesis, immunosuppression, and cancer progression in the inflammatory environment and alkaline conditions.

To ensure survival and proliferation, cancer cells fight against exogenous or endogenous stress with stress response pathways. The ability of stress-responsive genes to dynamically and non-genetically adapt to different stresses and respond to changes in TME is the primary hallmark of metastasis [22]. Cellular plasticity is important in cancer progression, where the same molecules and pathways play different roles at different steps during proliferation and metastatic dissemination [23]. The primary markers of metastasis are gene expression regulation, metabolic adaptation, and plasticity, namely the ability to dynamically nongenetically adapt to different pressures [24]. Our RNA-seq results identified early growth response 1 (EGR1) as a differentially expressed gene. EGR1 plays a dual role in regulating oxidative stress. Mitochondria are the stress response elements of cells, and the information exchange between the endoplasmic reticulum (ER) and mitochondria can affect the fate of cells. The ER provides calcium ions to mitochondria through the mitochondria-associated ER membrane [25]. When ER stress occurs, a large amount of calcium ions flow from the ER to mitochondria, which causes excessive calcium ion load in mitochondria and accelerates apoptosis [26]. EGR1 silencing decreases oxidative stress, while its overexpression has the opposite effects.

Tumors in a full EMT state invade surrounding tissue as single mesenchymal cells, whereas hybrid EMT states promote the collective migration of cells, with tumor cells at the leading edge presenting a more pronounced EMT phenotype compared with follower cells [27]. Hybrid EMT is associated with plasticity, stemness, invasiveness, and enhanced metastatic ability. Keratin 7 (KRT7), a member of the keratin family, is abnormally expressed in various types of cancer and promotes metastasis [28]. KRT7 regulates EMT and cell matrix adhesion through integrin-β1-focal adhesion kinase signaling. Our results suggested that KRT7 may be a potential molecular marker for prognosis prediction in patients with GC.

Surgical resection is a common treatment approach for GC. By removing the diseased tissue, the survival of patients can be prolonged. However, as a result of the weakened immune function of tumor patients, invasive operations can lead to pathogen infections, including infection by *S. aureus*, *E. coli*, and Pseudomonas aeruginosa [29]. Abdominal cavity infection is a common complication after GC surgery and can prolong the postoperative recovery time and hospital stay of patients. Studies have shown that abdominal cavity infection may also reduce the 5-year survival rate and recurrence-free survival rate of GC patients after surgery [30]. Abdominal cavity infection not only delays the recovery of gastrointestinal function after surgery but also induces local immune suppression in severe cases, increases the risk of tumor recurrence, and can be life threatening. In our study, co-cultivation of Mg alloys and pathogenic bacteria (*E. coli* and *S. aureus*) revealed that Mg alloys inhibited bacterial growth. These results indicate that Mg alloys have broad application prospects in inhibiting bacteria. Preparing such materials into antibacterial coatings may effectively reduce incidences of iatrogenic infections and improve the prognosis and quality of life of patients.

Mg alloys have various properties, including biodegradability, biocompatibility, and mechanical properties, and show great potential for application in implantable instruments such as stents, screws, and staples [31,32]. By inhibiting the activity of various enzymes and altering membrane permeability and mitochondrial function, Mg alloys also inhibit the growth of tumor cells; however, the specific mechanism has not been clear. In our study, we optimized processing technology and changed the microstructure of materials to obtain Mg alloys with excellent mechanical properties and stable degradation, with the aim for using Mg alloys in the clinical application of anastomosis nails. Our study results elucidate the influence of Mg alloys on GC and suggest that Mg alloys may be used as a scaffold and in anastomotic nails to inhibit surviving tumor cell invasion and metastasis into surrounding tissues.

This study has several limitations. Under continuous selective pressure, the formation of metastatic cancer subclones in tumors may be promoted. Cells with migratory potential are gradually selected during treatment, accompanied by a dynamic cycle of dedifferentiation and redifferentiation, which endows cells with stronger phenotypic plasticity and environmental adaptability. Future studies using single-cell transcriptome analysis are required to more directly reveal the molecular characteristics of heterogeneous subpopulations and their dynamic evolution patterns during the metastasis process. We only examined two common pathogenic bacteria in the current study. To further expand the breadth and application potential of the research, additional work is required to obtain a more systematic scope of antibacterial activity, and long-term toxicity assessment is necessary. The results of such studies will help provide a scientific basis for the development of new clinical validation.

## 5. Conclusions

Mg alloys, biodegradable biomaterials, represent a promising therapeutic resource for GC. Our study confirms that Mg alloys not only effectively suppressed GC tumor cell proliferation but also significantly reduced GC tumor volume in tumor-bearing mice, leading to progressive ablation of tumor tissue. These new findings demonstrate that Mg alloys serve as a novel therapeutic resource for GC, which may overcome the limitations of traditional therapies in terms of therapeutic persistence and safety. These materials have important scientific significance and clinical translational value for improving the treatment of patients with GC.

## Figures and Tables

**Table 1:** Primer pairs used for real-time PCR.

Gene	Forward	Reverse
MMP1	AAAATTACACGCCAGATTTGCC	AAAATTACACGCCAGATTTGCC
IL11	GACCTACTGTCCTACCTGCG	AGTCTTCAGCAGCAGCAGTC
COL5A3	AACAAGGAAATTTGGACCTCAA	GAGTCCGAGATGGATATTCTGC
SLC6A12	CCTGGCCACTTTCCTCTTCTC	CAGGAACCAGCCAATGGAGTA
EGR1	GGTCAGTGGCCTAGTGAGC	GTGCCGCTGAGTAAATGGGA
CAV1	AGCAAAAGTTGTAGCGCCAG	GACCACGTCGTCGTTGAGAT
CFLAR	GGACTTGGCTGAACTGCTCTAC	TCCAAATCCTCACCAATCTCTG
IFRD1	TGCAGTGGTTATAGCGATCCT	CCTTGTCTTCGCACTCTTATCC
CASC19	CTCAGCATTTGCCATACTACAT	TTCTAAC-CCAGGCACTCCAA
KRT7	TCCGCGAGGTCACCATTAAC	GCTCTGTCAACTCCGTCTCAT
GAPDH	GAAGGTGAAGGTCGGAGTC	GAAGATGGTGATGGGATTTC

MMP1—matrix metallopeptidase 1; IL11—interleukin 11; COL5A3—collagen type V alpha 3 chain; SLC6A12—solute carrier family 6 member 12; EGR1—early growth response 1; CAV1—caveolin 1; CFLAR, CASP8, and FADD-like apoptosis regulator; IFRD1—interferon-related developmental regulator 1; CASC19—cancer susceptibility 19; KRT7—keratin 7; GAPDH—glyceraldehyde-3-phosphate dehydrogenase.

**Table 2:** The chemical composition of Mg alloys.

Terms	No Immersion		Hank’s Liquid Immersion				
**Element**	**Magnesium**	**Zirconium**	**Magnesium**	**Zirconium**	**Calcium**	**Aluminium**	**Oxygen**
AN	12	40	12	40	20	13	8
norm. C [wt.%]	86.94	13.06	18.20	26.74	7.03	1.98	46.05
Atom. C [at.%]	96.15	3.85	17.96	7.03	4.21	1.76	69.04

## Data Availability

The original contributions presented in the study are included in the article, further inquiries can be directed to the corresponding author.

## Abbreviations

GC—gastric cancer; Mg—Magnesium; UV—ultraviolet; RTV—relative tumor volume; GO—gene ontology; KEGG—Kyoto Encyclopedia of Genes and Genomes; CFU—colony forming units; SEM—scanning electron microscopy; MMP1—matrix metallopeptidase 1; IL11—interleukin 11; COL5A3—collagen type V alpha 3 chain; SLC6A12—solute carrier family 6 member 12; EGR1—early growth response 1; CAV1—caveolin 1; CFLAR, CASP8 and FADD-like apoptosis regulator; IFRD1—interferon-related developmental regulator 1; CASC19—cancer susceptibility 19; KRT7—keratin 7; GAPDH—glyceraldehyde-3-phosphate dehydrogenase; BHI—Brian Heart Infusion; TME—tumor microenvironment; ER—endoplasmic reticulum; MAM—mitochondria-associated ER membrane; UPR—unfolded protein response; ROS—reactive oxygen species; OXPHOS—oxidative phosphorylation.
